# Camphor and Eucalyptol—Anticandidal Spectrum, Antivirulence Effect, Efflux Pumps Interference and Cytotoxicity

**DOI:** 10.3390/ijms22020483

**Published:** 2021-01-06

**Authors:** Marija Ivanov, Abhilash Kannan, Dejan S. Stojković, Jasmina Glamočlija, Ricardo C. Calhelha, Isabel C. F. R. Ferreira, Dominique Sanglard, Marina Soković

**Affiliations:** 1 Department of Plant Physiology, Institute for Biological Research “Siniša Stanković”—National Institute of Republic of Serbia, University of Belgrade, Bulevar Despota Stefana 142, 11000 Belgrade, Serbia; dejanbio@yahoo.com (D.S.S.); jasna@ibiss.bg.ac.rs (J.G.); mris@ibiss.bg.ac.rs (M.S.); 2 Institute of Microbiology, University Hospital Lausanne and University Hospital Center, Rue du Bugnon 48, 1011 Lausanne, Switzerland; abhilifescizurich@gmail.com (A.K.); Dominique.Sanglard@chuv.ch (D.S.); 3 Centro de Investigação de Montanha (CIMO), Instituto Politécnico de Bragança, Campus de Santa Apolónia, 5300-253 Bragança, Portugal; calhelha@ipb.pt (R.C.C.); iferreira@ipb.pt (I.C.F.R.F.)

**Keywords:** terpenoids, camphor, eucalyptol, antifungal activity, virulence factors, efflux pumps, cytotoxicity

## Abstract

*Candidaalbicans* represents one of the most common fungal pathogens. Due to its increasing incidence and the poor efficacy of available antifungals, finding novel antifungal molecules is of great importance. Camphor and eucalyptol are bioactive terpenoid plant constituents and their antifungal properties have been explored previously. In this study, we examined their ability to inhibit the growth of different *Candida* species in suspension and biofilm, to block hyphal transition along with their impact on genes encoding for efflux pumps (*CDR1* and *CDR2*), ergosterol biosynthesis (*ERG11*), and cytotoxicity to primary liver cells. Camphor showed excellent antifungal activity with a minimal inhibitory concentration of 0.125–0.35 mg/mL while eucalyptol was active in the range of 2–23 mg/mL. The results showed camphor’s potential to reduce fungal virulence traits, that is, biofilm establishment and hyphae formation. On the other hand, camphor and eucalyptol treatments upregulated *CDR1;* *CDR2* was positively regulated after eucalyptol application while camphor downregulated it. Neither had an impact on *ERG11* expression. The beneficial antifungal activities of camphor were achieved with an amount that was non-toxic to porcine liver cells, making it a promising antifungal compound for future development. The antifungal concentration of eucalyptol caused cytotoxic effects and increased expression of efflux pump genes, which suggests that it is an unsuitable antifungal candidate.

## 1. Introduction

*Candida albicans* resides as a part of the healthy human microbiome, however, it is also one of the most frequent human fungal pathogens [1,2]. Due to the fact that mortality rates in patients suffering from candidemia can be up to 54% [3], this fungus represents a serious risk to human health and a significant economic burden for our societies. Therefore, the search for novel alternatives for candidiasis therapy is of major interest. This has been highlighted in several recent review papers [4,5,6] that provide a contemporary overview of the current knowledge on alternative antifungal therapies.

The pathogenicity of *C. albicans* is directly related to the expression of various virulence factors that this fungus uses to damage the host cell. These include the transition from yeast to the hyphal growth phase, biofilm formation and the secretion of hydrolytic enzymes [7]. Virulence factors are now being extensively studied as antifungal targets since commercial antifungals are mostly ineffective against them, making the search for efficient fungal antivirulence agents an attractive and challenging mission [6,8,9]. Besides using an antivirulence approach as a novel antimicrobial strategy, several authors [10,11,12] have also suggested the inhibition of microbial efflux pumps as an alternative strategy for combating microbial pathogens. Some of the fungal efflux pumps, such as the ones belonging to ATP binding cassette transporters, Cdr1 and Cdr2, have been proven to have a role in the development of resistance to azole drugs [13]. Likewise, in the case of patients suffering from fungal infections, upregulation of *CDR1* and *CDR2* is an undesirable property for any therapeutic given along the way [14], while their downregulation is seen as a promising antifungal trait [15]. *ERG11* is another gene whose expression is linked to antifungal resistance. This gene is involved in the biosynthesis of ergosterol, an essential lipid in the fungal kingdom; *ERG11* upregulation in *C. albicans* leads to azole treatment insensitivity [16].

Many different compounds of natural origin have been explored so far [17,18,19] in order to shed light on the huge antifungal potential of natural products. Terpenoids and terpenes are one of the most abundant classes of compounds found in nature, with antimicrobial activity being one of the many bioactivities attributed to them [20,21]. In the current study, camphor and eucalyptol were selected as representatives of terpenoid compounds in order to examine their anticandidal potential. This study focused on the ability of the terpenoids to inhibit the growth of different *Candida* species in suspension and biofilm; to block hyphal transition along with their impact on genes encoding for efflux pumps (*CDR1* and *CDR2*); their effect on ergosterol biosynthesis (*ERG11*); and their cytotoxicity to primary liver cells with the aim to enlighten novel antifungal strategy.

## 2. Results

### 2.1. Impact of Camphor and Eucalyptol on Candida Albicans Growing in Planktonic and Biofilm Forms

Of the two compounds that were subject to this investigation, camphor was found to have much better antifungal potential against all the examined *Candida* strains compared to eucalyptol (minimal inhibitory concentration (MIC) 0.125–0.35 mg/mL and 2–23 mg/mL, respectively) (Table 1). Strains showed different susceptibility to camphor with the most sensitive strains being *C. albicans* 475/15, *C. albicans* 527/14, *C. albicans* 10/15, *C. albicans* 532/15, *C. albicans* 16/15 and *C. parapsilosis* ATCC 22019 (MIC 0.125 mg/mL), while *C. krusei* H1/16 was most resistant to the camphor treatment (MIC 0.35 mg/mL). On the other hand, eucalyptol resulted in the strongest inhibition of *C. glabrata* 4/6/15 and *C. parapsilosis* ATCC 22019 (MIC 2 mg/mL), while much lower activity was observed in the rest of the examined microorganisms, especially *C. albicans* 475/15, *C. albicans* 527/14 and *C. albicans* 10/15 with MIC 23 mg/mL.

**Figure 1:**
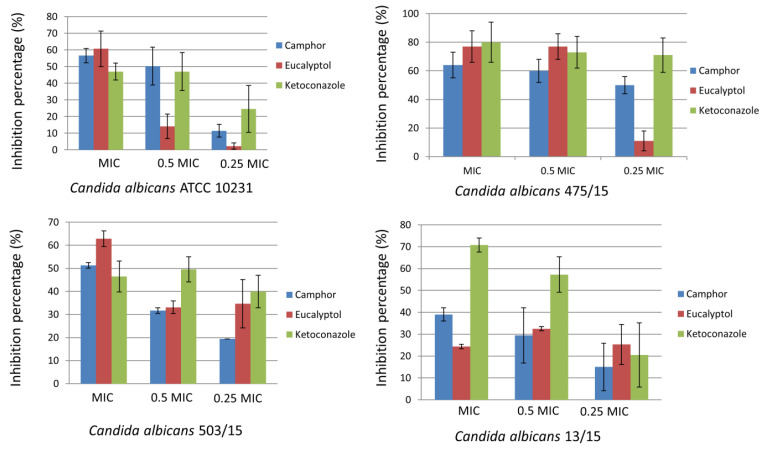
Inhibition of *Candida albicans* biofilm formation after treatment with camphor, eucalyptol and ketoconazole, expressed as inhibition percentage (100% means no biofilm was established), values represent means ± SD of three replicates.

Treatment with both of the terpenoids impaired the ability of various *C. albicans* strains to establish biofilms in vitro (Figure 1). The application of camphor and eucalyptol reduced the formation of biofilm biomass by more than 50% in three *C. albicans* strains (*C. albicans* ATCC 10231, *C. albicans* 475/15 and *C. albicans* 503/15) at their MIC concentrations. Biofilm formation of *C. albicans* ATCC 10231 and *C. albicans* 475/15 was also inhibited (>50% inhibition) with camphor in a concentration equal to its ½ MIC (Figure 1). Since applied MIC and subMIC concentrations of camphor were lower than those for eucalyptol (Table 1), its antibiofilm potential is of higher significance. 

**Figure 2:**
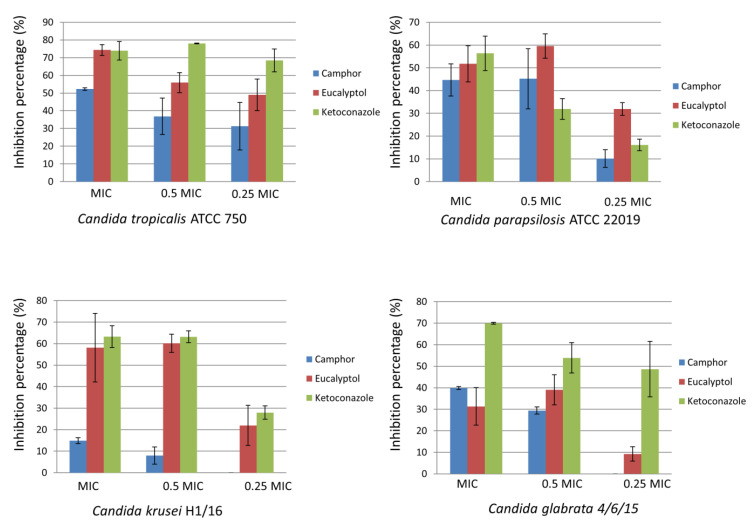
Inhibition of non-albicans *Candida* biofilm formation after treatment with camphor, eucalyptol and ketoconazole, expressed as inhibition percentage (100% means no biofilm was established), values represent means ± SD of three replicates.

Among the examined non-albicans *Candida* strains, biofilm establishment was significantly disrupted for the strain *C. tropicalis* ATCC 750 at the MICs of both camphor and eucalyptol (>50% inhibition). On the other hand, *C. krusei* H1/16 and *C. glabrata* 4/6/15 biofilms were the strains most resistant to the application of camphor and eucalyptol, respectively (Figure 2).

### 2.2. Camphor and Eucalyptol as Inhibitors of Candida Albicans Hyphae Formation

**Figure 3:**
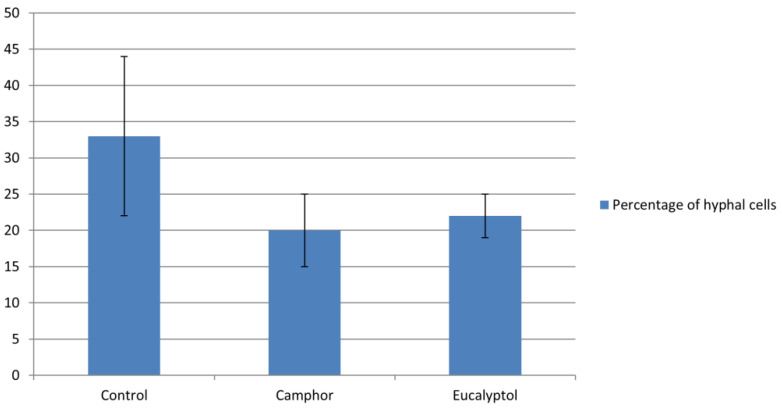
The number of hyphae and germ tubes was determined 4 h post treatment of *Candida albicans* cells with compounds and the percentage of hyphal cells was calculated. Values represent means ± SD of three replicates.

The application of camphor (0.125 mg/mL) induced a notable reduction in the number of hyphal cells as shown in Figure 3, similar to eucalyptol applied in higher concentration (23 mg/mL). 

**Figure 4:**
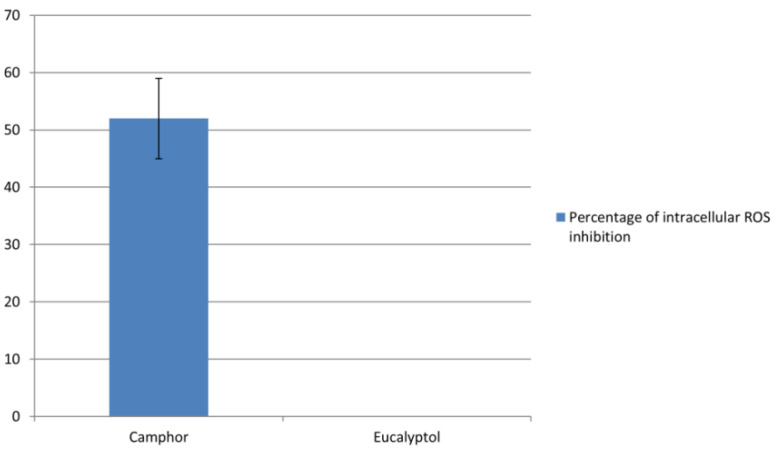
Percentage of inhibition of reactive oxygen species in *Candida albicans* cells treated with camphor and eucalyptol (no activity). Values represent means ± SD of three replicates.

Hyphal growth and damage of the host epithelial cells is linked to the increase in reactive oxygen species (ROS) generated by *C. albicans* [22], thus, the ability of camphor to reduce ROS generation by 52% is of great interest in the development of antivirulent candidiasis therapy. On the other hand, eucalyptol did not reduce the generation of ROS (Figure 4).

### 2.3. Impact on Genes Coding Fungal Efflux Pumps and Gene Involved in Ergosterol Biosynthesis

**Figure 5:**
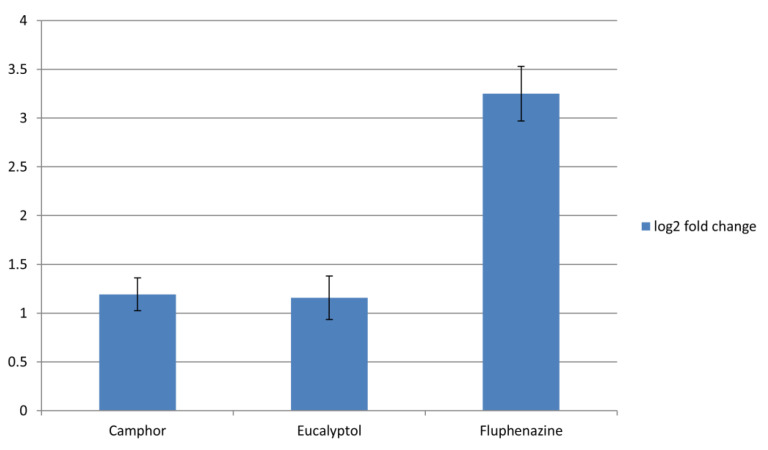
Expression levels of *CDR1* after treatment with MIC of camphor and eucalyptol; fluphenazine was used as a positive control for *CDR1* expression. Values are expressed as the log2 fold change (log2 FC) of Relative Quantification (RQ) values and presented as an average of two biological replicates.

Treatment with both camphor and eucalyptol led to the increased expression of *CDR1* (log2 fold change (FC) > 1) in fungal cells (Figure 5).

**Figure 6:**
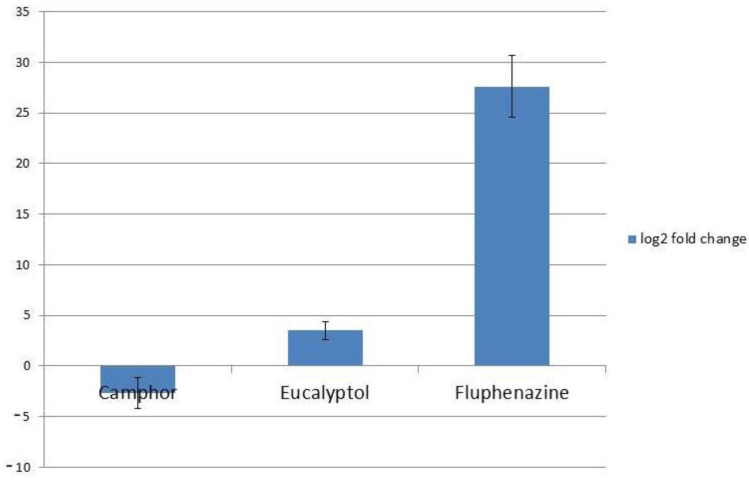
Expression levels of *CDR2* after treatment with MIC of camphor and eucalyptol; fluphenazine was used as a positive control for *CDR2* expression. Values are expressed as the log2 FC of RQ values and presented as an average of two biological replicates.

On the other hand, the two investigated compounds had a different impact on *CDR2* expression—treatment with camphor reduced the level of *CDR2* (log2FC < −2) while treatment with eucalyptol upregulated this gene (log2FC > 3) (Figure 6).

**Figure 7:**
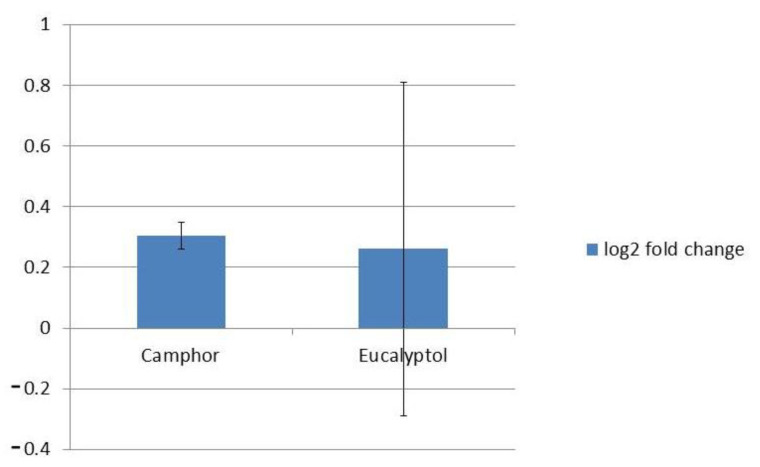
Expression levels of *ERG11* after treatment with MIC of camphor and eucalyptol. Values are expressed as the log2 FC of RQ values and presented as an average of two biological replicates.

The application of the selected compounds in the current study did not interfere with the *ERG11* expression (log2FC < 0.5) (Figure 7).

### 2.4. Camphor and Eucalyptol—Diverse Cytotoxicity to Porcine Liver Cells

Camphor did not influence porcine liver cell proliferation at the maximum tested concentration (400 µg/mL, Table 2). Eucalyptol exhibited cytotoxicity with GI_50_ 56 µg/mL (Table 2), a concentration much lower than the one required to inhibit fungal growth (Table 1). These results showed that camphor was much safer than eucalyptol since it had no toxic effects on tested cells and can be used at higher concentrations without side effects on the PLP cells. Nevertheless, more studies are needed to corroborate these results, especially in vivo experiments.

## 3. Discussion

Previous studies focused on camphor oil have determined its MIC and MFC as 0.5% and 1%, respectively, for a range of *C. albicans* strains [23] and as 0.5% (*w*/*v*) for *C. albicans* DAY185 [24]. On the other hand, a study by Zuzarte et al. [25] claimed that camphor is not efficient in reducing the growth of *C. albicans* ATCC 10231 and clinical isolates. Previous studies of the anticandidal potential of eucalyptol have reported MIC 8 g/L and MFC 64 g/L [26], MIC ≥ 8% (*v*/*v*) [27] and MIC 4 mg/mL [28]. In their search for *C. albicans* inhibitors, Bin Jantan et al. [29] found that the activity of eucalyptol was lower than camphor (MIC > 5 µg/µL and 3.75 µg/µL, respectively). Furthermore, essential oil of *Salvia officinalis* has shown remarkable antifungal potential against *C. albicans* (MIC 2.5 µL/mL) [30], suggesting that a synergistic activity between its dominant compounds, camphor and eucalyptol, rather than its single constituents contribute to this effect. Aromatic plant compounds, camphor, carvacrol and eucalyptol were tested in a study by Sokovic et al. [31] and of these, eucalyptol exhibited the lowest antifungal potential against different pathogenic fungal species; it was also less effective compound in tea tree oil [32]. In our study, the antifungal potential of eucalyptol was also proven to be much lower than the potential exhibited by camphor (Table 1). This is the first comparative study of their antifungal and antibiofilm potential in a range of oral clinical isolates. Furthermore, to the best of the authors’ knowledge, this is the first study to highlight the effect of selected compounds on the expression of genes encoding for *C. albicans* efflux pumps.

The antibiofilm activities of camphor (0.005 and 0.01% *w*/*v*) were previously confirmed by Manoharan et al. [24] on *C. albicans* strain DAY185. It has been shown that the mechanism of camphor antibiofilm action involves downregulation of *HWP1* (hyphal wall protein 1), *RBT1* (repressed by Tup1) and *EED1* (epithelial escape and dissemination 1) [24]. A study by Sancineto et al. [33] investigated camphor diselenide and found it was efficient against both candidal and bacterial biofilms when applied in concentrations of 6.25–50 mg/L, which suggests that some modifications of this compound could significantly increase its bioactivity. The potential of camphor to interfere with fungal biofilms could be further explored in order to find novel antivirulent agents and develop them as a part of an antifungal strategy. Future directions might also include synthetizing novel camphor derivatives in order to improve its bioactivities [33].

In a study by Hendry et al. [26], eucalyptol was established as a more efficient antibiofilm agent than eucalyptus oil. The eucalyptol MIC for *C. albicans* (ATCC 76615) cells embedded in biofilms was two times lower than the MIC for the cells in suspension (4 g/L compared to 8 g/L) [26]. In another study, application of 1/16 MIC of eucalyptol was able to significantly interfere with MRSA (methicillin resistant *Staphylococcus aureus*) biofilm development [34]. Although the potency of eucalyptol to inhibit fungal [26] and bacterial [34] biofilms was determined previously, due to its high MIC (Table 1) observed in our study it is necessary to expand the microbial strains used in these assays in order to completely elucidate its antimicrobial and antibiofilm capacity. 

Complete *C. albicans* hyphal inhibition with 0.01% camphor was noticed by Manoharan et al. [24] along with reduced expression of *ECE1* (extent of cell elongation), a hypha-specific gene [24]. The application of eucalyptol (23 mg/mL) also reduced the number of hyphal cells (Figure 3), while significantly lower concentrations of this compound (1 mg/mL) provided anti-hyphal activity in a previous study against hyphal formation of the reference strain *C. albicans* ATCC 90028 [28].

Unlike fungal cells (Figure 4), treatment of rat thymocytes with camphor significantly induced the generation of ROS [35]. On the other hand, eucalyptol was not able to inhibit ROS production in fungal cells (Figure 4), while it inhibited ROS generation in a human astrocytoma cell line treated with hydrogen peroxide and acted as a regulator of redox balance inside cells [36]. Differences between fungal and human cells, including lack of cell wall in human cells [37], might be the reasons for these diverse consequences of eucalyptol application in human and fungal cells. 

Eucalyptol was previously shown to inhibit bacterial efflux pumps in the *Pseudomonas aeruginosa* and *Acinetobacter baumannii* strains [38], so, observed stimulation of efflux pumps gene expression (Figure 5) might be specific to fungal cells. The increased expression of genes encoding for efflux pumps is not a desirable trait of potential antifungal agents because it leads to requiring higher doses of antifungals associated with harmful side effects [6,39]. Bearing that in mind, the efflux-inducing properties of camphor and eucalyptol determined in our study and their potential adverse effect on human health should be further explored. 

The impact of camphor and eucalyptol on expression levels of *CDR2* was explored as well. The observed effect of these compounds on *CDR2* expression levels (Figure 6) implies that these two compounds might affect different regulators of *CDR* gene expression. *TAC1* (transcriptional activator of *CDR* genes) is an activator of transcription included in the regulation of both *CDR1* and *CDR2*, since both genes have Tac1 binding regulatory element DRE (drug-responsive element) [40], which suggests it as a possible eucalyptol target. Regulation of *CDR1* expression also involves the CaNdt80p transcription factor [41]; there is no data on whether this factor also regulates *CDR2*, thus it might be one of camphor’s specific targets in *C. albicans* cells; however, this hypothesis should be further investigated. In a previous study, the application of thymol and carvacol at their minimal inhibitory concentrations reduced efflux in a fluconazole-resistant *Candida* strain by 70–90% via a mechanism involving the decreased expression of efflux pump genes *CDR1* and *MDR1* [42]. On the other hand, bacterial efflux pumps are efficiently inhibited with terpene geraniol [43] and terpenoid ursolic acid [44], although not much is recorded about the undesirable trait of molecules observed in this study—efflux pump induction. Given that treatment of *C. albicans* cells with eucalyptol leads to the upregulation of both *CDR1* and *CDR2*, special attention should be given to the application of this compound in pharmaceuticals, especially in combination with azoles, since it might reduce their activity.

Since the *ERG11* gene encodes the azole target, CYP51, application of any compound that might increase *ERG11* expression could lead to azole resistance [45]. The antifungal activity of different essential oils with high terpenoid and terpene content has previously been linked to reduction in ergosterol content [46,47,48]. On the other hand, the study by Połeć et al. [49] found that phytosterols, rather than fungal sterols, are influenced by eucalyptol and terpinen-4-ol. In this study we did not observe any significant interference with the *ERG11* gene when selected terpenoids were applied (Figure 7).

One of the crucial steps at the very beginning of the development of novel antifungals is to observe the effects that the investigated compounds might have on cultured mammalian cells [50]. A previous study showed that camphor influenced the proliferation of fetal lung fibroblasts MRC-5 with IC_50_ 11.0 mM [51]. In the study by Nikolic et al. [51], eucalyptol showed IC_50_ 11 mM against fetal lung fibroblasts MRC-5, while both 0.025 and 0.05% eucalyptol induced significant cytotoxicity against peritoneal macrophages in a study by Zaccaro et al. [52]. The toxicity of eucalyptol that was determined in our study once again raises questions regarding its safety for human use and puts it out of contention for potential antifungal applications.

## 4. Materials and Methods 

### 4.1. Microbial Culture Conditions

Strains used in the study were clinical isolates: *C. albicans* 475/15, *C. albicans* 527/14, *C. albicans* 10/15, *C. albicans* 27/15, *C. albicans* 532/15, *C. albicans* 503/15, *C. albicans* 13/15, *C. albicans* 16/15, *C. krusei* H1/16 (*Pichia kudriavzevii*), *C. glabrata* 4/6/15 as well as strains obtained from the American Type Culture Collection: *C. albicans* ATCC 10231, *C. tropicalis* ATCC 750 and *C. parapsilosis* ATCC 22019. Clinical *Candida* strains were isolated from oral cavities of patients at the ENT Clinic, Clinical Hospital Centre Zvezdara, Belgrade, Serbia and determined by using CHROMagar (Biomerieux, Craponne, France) and on HiCrome™ Candida differential agar plates (HiMedia, Mumbai, India). Fungal strains are deposited at the Mycological Laboratory, Department of Plant Physiology, Institute for Biological Research ‘‘Siniša Stanković’’—National Institute of Republic of Serbia, University of Belgrade.

### 4.2. Microdilution Method

Microdilution assay [53] was used to determine anticandidal activity in 96-well microtiter plates, with some modification. Yeast cultures were adjusted to 1.0 × 10^5^ CFU/well with sterile saline. The 96-well microtiter plates were incubated with serially diluted compounds at 37 °C for 24 h after which minimal inhibitory concentration (MIC) and minimal fungicidal concentration (MFC) were determined. The lowest concentrations without microscopically observed growth were considered as the MIC, while the MFC values were determined as the concentrations without visible growth after serial sub-cultivation of 10 µL of samples in 100 µL of broth/well at 37 °C for 24 h. Ketoconazole (SigmaAldrich, Darmstadt, Germany) was used as a positive control. Camphor and eucalyptol were obtained from SigmaAldrich, Steinheim, Germany.

### 4.3. Antibiofilm Activity

The influence of selected compounds on the ability of *C. albicans* ATCC 10231, *C. albicans* 475/15, *C. albicans* 503/15, *C. albicans* 13/15, *C. krusei* H1/16, *C. glabrata* 4/6/15, *C. tropicalis* ATCC 750 and *C. parapsilosis* ATCC 22,019 to form biofilms was investigated as previously described [54]. Yeasts were incubated with the compounds in their previously determined concentrations equal to MIC, 0.5 MIC and 0.25 MIC in 96-well microtiter plates with an adhesive bottom (Sarstedt, Nümbrecht, Germany), at 37 °C for 24 h. After incubation, wells were washed twice with sterile PBS (Phosphate buffered saline, pH 7.4) and methanol was added into each well. After the fixation of the fungal cells, the methanol was discarded and the plate was air dried. Formed biofilms were stained with 0.1% crystal violet (Bio-Merieux, Craponne, France) for 30 min. The plate was washed with water and air dried. Ethanol 96% (Zorka Pharma—Hemija, Sabac, Serbia) was used to dissolve the biofilm bound stain. Absorbance was read on a Multiskan™ FC Microplate Photometer, Thermo Scientific™. The percentage of inhibition of biofilm formation was calculated according to the formula: ((A620control − A620sample)/A620control) × 100.

### 4.4. Inhibition of Morphological Transition

*C. albicans* 475/15 cells were incubated with the MICs of the tested compounds in YPD + 10% FBS at 37 °C for 4 h. Fungal cells were watched under microscope (Nikon Eclipse TS2, Amsterdam, The Netherlands) and the number of cells growing in yeast or hyphal and germ tube formations was determined. The assay was performed in triplicate and the percentage of hyphal cells was determined.

### 4.5. Determination of Intracellular ROS Levels in C. albicans 475/15

The impact of compounds on intracellular levels of ROS was determined according to Paez et al. [55]. *C. albicans* 475/15 was incubated with MICs of compounds overnight at 37 °C. The suspension of *C. albicans* treated cells (0.4 mL) was further incubated with 0.5 mL of nitro blue tetrazolium (1 mg/mL) at 37 °C for 30 min. After the addition of 0.1 mL 0.1 M HCl, tubes were centrifuged at 2500× *g* for 10 min. The pellets were treated with 0.6 mL dimethyl sulfoxide and 0.8 mL phosphate saline buffer and absorbance was recorded at 575 nm (Agilent/HP 8453 UV-Visible Spectrophotometer; Agilent Technologies, Santa Clara, CA, USA).

### 4.6. RNA Isolation and cDNA Synthesis

Total RNA was extracted from 5 mL *C. albicans* 475/15 logarithmic-phase cultures grown in YEPD medium by a technique of cell mechanical disruption with glass beads according to Sanglard et al. [56] and modified as described in Ivanov et al. [57]. The concentration and purity of the RNA was determined using a NanoDrop (ND-1000, Witec AG, Sursee, Switzerland) where OD260 nm/OD280 nm of the samples ranged from 1.80 to 2.05 and the OD260 nm/OD230 nm ranged from 2.00 to 2.60. For qPCR assay, 1 µg RNA was reverse transcribed to cDNA (Transcriptor High Fidelity cDNA synthesis kit, Roche) using random hexamer as a priming method. Prior to reverse transcription reaction, the total RNA samples were treated with DNase for 30 min at 37 °C according to the manufacturer’s instructions (DNA-free™ DNA Removal Kit, Ambion, Bleiswijk, The Netherlands).

### 4.7. qPCR

For the qPCR assay we used primers (0.2 µM) and probes (0.2 µM) for *ACT1*, *CDR1*, *CDR2* and *ERG11* genes (Table 3). Assay was performed by StepOnePlusTM Real Time PCR System using the iTAQ Supermix with ROX (BioRad, Reinach, Switzerland) according to the manufacturer’s instructions. Normalization of expression was done with *ACT1*, and fold changes were calculated for *CDR1, CDR2* and *ERG11* in vitro in the absence and presence of the compounds in their previously determined minimal inhibitory concentrations for 30 min.

### 4.8. Cytotoxicity of Compounds to Porcine Liver Primary Cells

Porcine liver was obtained from a local slaughterhouse, freshly harvested, and subsequently exploited for the preparation of PLP2 cell line [58,59]. The obtained tissue of the liver was washed in Hank′s balanced salt solution enriched with 100 U/mL penicillin and 100 μg/mL streptomycin and was separated into explants (1 × 1 mm^3^). Some of the explants were transferred into 25 cm^2^ tissue flasks in Dulbecco′s modified Eagle′s medium (DMEM) enriched with 10% FBS, 2 mM nonessential amino acids, 100 U/mL penicillin, and 100 mg/mL streptomycin. Explants were placed in a humidified atmosphere containing 5% CO_2_ at 37 °C with fresh medium added every 2 days. Direct observation of the cells was conducted each 2–3 days with a phase-contrast microscope. Before confluence was achieved, cells were sub-cultured and 1.0 × 10^4^ cells per well was seeded in 96-well plates and cultivated in DMEM with 10% FBS, 100 U/mL penicillin, and 100 μg/mL streptomycin. In order to determine the cytotoxicity, we used the previously described Sulforhodamine B assay [59]. The cytotoxicity results were defined by GI_50_ values corresponding to the concentration of compound that inhibits 50% of the net cell growth. The positive control in this assay was the cytotoxic agent, ellipticine.

### 4.9. Statistical Analysis

The results are presented as the mean value of three replicates ± standard deviation (SD). The data were analyzed by one-way analysis of variance (ANOVA) followed by Tukey′s HSD test with *α* = 0.05 with the SPSS v. 18.0 program. QPCR analysis was performed in technical triplicates and the results are presented as the mean value of two biological replicates.

## 5. Conclusions

In this study, camphor and eucalyptol, terpenoids that are abundantly present in a range of medicinal plants, showed antifungal properties. Although the anticandidal potential of these terpenoids has been studied previously with a focus on the determination of MICs and MFCs, this is the first parallel study of their inhibitory activity with regard to 13 *Candida* strains, including 8 different *C. albicans* oral isolates reporting the involvement of terpenoids on the expression of efflux pumps in treated yeast. However, eucalyptol antimicrobial potential was achieved in concentrations that are toxic to liver cells and induce the expression of genes encoding fungal efflux pumps, suggesting it is not suitable for further drug development. On the other hand, the activities of camphor in terms of its antifungal potential were more promising. Camphor showed better antimicrobial, antibiofilm and antihyphal potential when compared to eucalyptol and all the above-mentioned bioactive properties could be fulfilled by quantities that were not toxic to liver cells. Camphor induced expression of the *CDR1* gene, while unlike eucalyptol, it downregulated *CDR2*. Due to its impact on efflux pumps it could be suggested that treatment with camphor along with azole therapy would induce less side effects than the application of eucalyptol. Our results confirm the great potential of camphor as an antifungal therapeutic and raise doubts regarding the safety of eucalyptol as an anticandidal molecule. Eucalyptol is already present in a wide range of pharmaceutical products so there is an urgent need to conduct further research regarding its potential interference with antifungal azole drugs.

## Figures and Tables

**Table 1:** Anticandidal activity of camphor and eucalyptol. Results are expressed in mg/mL. Values are expressed as means ± SD of three replicates. Different letters (a, b, c) in each row indicate a significant statistical difference between the samples (*p* < 0.05). MIC and MFC values of the compounds are compared separately for each of the fungal strain tested.

Strain	Camphor		Eucalyptol		Ketoconazole	
	MIC	MFC	MIC	MFC	MIC	MFC
*C. albicans* 475/15	0.125 ± 0.01 ^b^	0.25 ± 0.02 ^b^	23 ± 0.1 ^c^	46 ± 0.2 ^c^	0.0031 ± 0.0001 ^a^	0.0062 ± 0.0001 ^a^
*C. albicans* 527/14	0.125 ± 0.01 ^b^	0.25 ± 0.02 ^b^	23 ± 0.2 ^c^	46 ± 0.2 ^c^	0.0031 ± 0.0001 ^a^	0.0062 ± 0.0001 ^a^
*C. albicans* 10/15	0.125 ± 0.02 ^b^	0.25 ± 0.02 ^b^	23 ± 0.1 ^c^	46 ± 0.2 ^c^	0.0031 ± 0.0001 ^a^	0.05 ± 0.0001 ^a^
*C. albicans* 27/15	0.25 ± 0.004 ^b^	0.5 ± 0.02 ^b^	6 ± 0.08 ^c^	12 ± 0.2 ^c^	0.0031 ± 0.001 ^a^	0.1 ± 0.01 ^a^
*C. albicans* 532/15	0.125 ± 0.01 ^b^	0.25 ± 0.01 ^b^	6 ± 0.08 ^c^	12 ± 0.2 ^c^	0.0031 ± 0.0001 ^a^	0.0062 ± 0.0001 ^a^
*C. albicans* 503/15	0.25 ± 0.008 ^b^	0.5 ± 0.02 ^b^	3 ± 0.06 ^c^	6 ± 0.08 ^c^	0.0031 ± 0.0001 ^a^	0.0062 ± 0.0001 ^a^
*C. albicans* 13/15	0.25 ± 0.01 ^b^	0.5 ± 0.008 ^b^	6 ± 0.08 ^c^	12 ± 0.2 ^c^	0.0016 ± 0.001 ^a^	0.05 ± 0.002 ^a^
*C. albicans* 16/15	0.125 ± 0.008 ^b^	0.25 ± 0.004 ^b^	6 ± 0.1 ^c^	12 ± 0.2 ^c^	0.0031 ± 0.001 ^a^	0.1 ± 0.001 ^a^
*C. albicans* ATCC 10231	0.175 ± 0.02 ^b^	0.35 ± 0.02 ^b^	4 ± 0.06 ^c^	8 ± 0.008 ^c^	0.0016 ± 0.001 ^a^	0.0062 ± 0.001 ^a^
*C. tropicalis* ATCC 750	0.175 ± 0.02 ^b^	0.35 ± 0.02 ^b^	4 ± 0.004 ^c^	8 ± 0.006 ^c^	0.0016 ± 0.002 ^a^	0.0062 ± 0.002 ^a^
*C. parapsilosis* ATCC 22019	0.125 ± 0.003 ^b^	0.25 ± 0.008 ^b^	2 ± 0.003 ^c^	4 ± 0.003 ^c^	0.0031 ± 0.0001 ^a^	0.0062 ± 0.0001 ^a^
*C. krusei* H1/16	0.35 ± 0.06 ^b^	0.7 ± 0.06 ^b^	4 ± 0.004 ^c^	8 ± 0.008 ^c^	0.0016 ± 0.001 ^a^	0.0032 ± 0.002 ^a^
*C. glabrata* 4/6/15	0.175 ± 0.02 ^b^	0.35 ± 0.04 ^b^	2 ± 0.004 ^c^	4 ± 0.007 ^c^	0.0016 ± 0.001 ^a^	0.0062 ± 0.002 ^a^

MIC—minimal inhibitory concentration, MFC—minimal fungicidal concentration.

**Table 2:** Cytotoxicity of camphor and eucalyptol (mean ± SD). GI_50_ values (µg/mL) corresponding to the sample concentration achieving 50% of growth inhibition in liver primary cultured PLP2 cells.

Compound	GI_50_
Camphor	>400
Eucalyptol	56 ± 4
Ellipticine	3.22 ± 0.2

**Table 3:** Sequences of TaqMan primers and probes used in qPCR.

**Primer**	**Sequence**
CDR1-ORF-F	ATGACTCGAGATATTTTGATA
CDR1-ORF-R	TTAACAGCAATGGTCTTTA
CDR2-ORF-F	TAGATATTTGAGCCACATG
CDR2-ORF-R	TTGGCATTGAAATTTTCG
ERG11-ORF-F	ATTGTTGAAACTGTCATTG
ERG11-ORF-R	CCCCTAATAATATACTGATCTG
ACT-ORF-F	GCATCACACTTTTTACAAT
ACT-ORF-R	AAACATAATTTGAGTCATCTTT
**Probe**	**Sequence**
CDR1-P2	CATTATGAGACCTGGTGAACTTACT
CDR2-P2	TTAGTCCATTCAACGGCAACATTAG
ERG11-P2	TTTGTCCCTTAGTGTTACACA
ACT1-P2	TTGCTCCAGAAGAACATCCAGT

## Data Availability

The data presented in this study are available in this article.

## Abbreviations

ROS	Reactive oxygen species
MIC	Minimum inhibitory concentration
MFC	Minimum fungicidal concentration
PBS	Phosphate buffered saline
FC	Fold change
